# Association between vitamins and risk of brain tumors: A systematic review and dose-response meta-analysis of observational studies

**DOI:** 10.3389/fnut.2022.935706

**Published:** 2022-07-29

**Authors:** Weichunbai Zhang, Jing Jiang, Yongqi He, Xinyi Li, Shuo Yin, Feng Chen, Wenbin Li

**Affiliations:** ^1^Department of Neuro-Oncology, Cancer Center, Beijing Tiantan Hospital, Capital Medical University, Beijing, China; ^2^College of Nursing, University of South Florida, Tampa, FL, United States

**Keywords:** vitamin, brain tumor, meta-analysis, β-carotene, folate, observational study

## Abstract

**Background:**

Brain tumor is one of the important causes of cancer mortality, and the prognosis is poor. Therefore, early prevention of brain tumors is the key to reducing mortality due to brain tumors.

**Objective:**

This review aims to quantitatively evaluate the association between vitamins and brain tumors by meta-analysis.

**Methods:**

We searched articles on PubMed, Cochrane Library, Web of Science, and Embase databases from inception to 19 December 2021. According to heterogeneity, the fixed-effects model or random-effects model was selected to obtain the relative risk of the merger. Based on the methods described by Greenland and Longnecker, we explored the dose-response relationship between vitamins and the risk of brain tumors. Subgroup analysis, sensitivity analysis, and publication bias were also used for the analysis.

**Results:**

The study reviewed 23 articles, including 1,347,426 controls and 6,449 brain tumor patients. This study included vitamin intake and circulating concentration. For intake, it mainly included vitamin A, vitamin B, vitamin C, vitamin E, β-carotene, and folate. For circulating concentrations, it mainly included vitamin E and vitamin D in the serum (25-hydroxyvitamin D and α-tocopherol). For vitamin intake, compared with the lowest intakes, the highest intakes of vitamin C (RR = 0.81, 95%CI:0.66–0.99, *I*^2^ = 54.7%, *P*_*for heterogeneity*_ = 0.007), β-carotene (RR = 0.78, 95%CI:0.66–0.93, *I*^2^ = 0, *P*_*for heterogeneity*_ = 0.460), and folate (RR = 0.66, 95%CI:0.55–0.80, *I*^2^ = 0, *P*_*for heterogeneity*_ = 0.661) significantly reduced the risk of brain tumors. For serum vitamins, compared with the lowest concentrations, the highest concentrations of serum α-tocopherol (RR = 0.61, 95%CI:0.44–0.86, *I*^2^ = 0, *P*_*for heterogeneity*_ = 0.656) significantly reduced the risk of brain tumors. The results of the dose-response relationship showed that increasing the intake of 100 μg folate per day reduced the risk of brain tumors by 7% (*P*_−*nonlinearity*_ = 0.534, RR = 0.93, 95%CI:0.90–0.96).

**Conclusion:**

Our analysis suggests that the intake of vitamin C, β-carotene, and folate can reduce the risk of brain tumors, while high serum α-tocopherol concentration also has a protective effect on brain tumors. Therefore, vitamins may provide new ideas for the prevention of brain tumors.

**Systematic Review Registration:**

PROSPERO, identifier CRD42022300683.

## Introduction

Brain tumors are the primary central nervous system tumors, with an annual incidence rate of 22.6/1,00,000 (1). They are a significant cause of cancer incidence rate and mortality, especially in children, accounting for 30% of cancer deaths (2). Because the prognosis of brain tumors, especially glioma, is poor, early prevention and detection are the keys to reducing brain tumor mortality (3).

Although the etiology of brain tumors had been studied for decades, the risk factors accounting for a large proportion of cases had not been found. In recent years, people had often paid attention to the relationship between diet and brain tumors. Some studies found that a diet rich in antioxidants, such as vegetables and fruits, could prevent brain tumors. Experimental studies had shown that these dietary antioxidants, could significantly inhibit the growth of cancer cells, especially brain tumor cells (4–6). Vitamins had a similar effect. Some vitamins with antioxidant properties, such as vitamin C and vitamin E, could inhibit tumor growth by eliminating free radicals and inducing apoptosis (7–9). In addition, fat-soluble vitamins, such as vitamin A and vitamin D, also played a certain preventive role by regulating cell differentiation and inhibiting cancer cell proliferation (9, 10). However, the current epidemiological results on vitamins and brain tumors were inconsistent. Chen et al. analyzed the diet of 236 patients with brain tumors through a case-control study and found that the intake of vitamin A was negatively correlated with the risk of glioma (odds ratio (OR) = 0.50, 95% confidence interval (95%CI):0.30–0.80) (11). However, Gile et al. arrived at the opposite conclusion (OR = 1.64, 95% CI:1.13–2.37) (12). A meta-analysis of seven articles showed that the highest intake of vitamin A in the diet was significantly associated with a reduced risk of glioma (relative risk (RR) = 0.80, 95% CI = 0.62-0.98, *P* = 0.014, *I*^2^ = 54.9%) (13). Tedeschi Blok et al. also found that people with a higher intake of carotene, the precursor of vitamin A, had a lower risk of brain tumors (OR = 0.72, 95% CI:0.54–0.98) (14), and vitamin C and vitamin E also had similar results in this study. However, Durrow et al. followed up with 545,770 participants for 7.2 years and found that dietary vitamin C (RR = 1.26, 95% CI: 0.96–1.66) and vitamin E (RR = 1.17, 95% CI:0.90–1.53) were not associated with the risk of brain tumors (15). Moreover, by detecting the concentrations of vitamin C and vitamin E in participants' serum, it was found that both had protective effects on brain tumors (vitamin C: OR = 0.19, 95% CI: 0.10–0.60, vitamin E: RR = 0.65, 95% CI:0.44–0.96) (16). In addition, the effect of folate on brain tumors had also attracted much attention. Studies had shown that both folate supplementation during pregnancy and children's high intake of folate could significantly reduce the risk of brain tumors (pregnant women OR = 0.60, 95% CI: 0.68–0.98, children: OR = 0.63, 95% CI: 0.41–0.97) (17).

Since the conclusions of previous studies were inconsistent, and most studies on the effects of vitamins on brain tumors included fewer cases, we quantitatively evaluated the relationship between various vitamin intake and *in vivo* exposure concentrations and brain tumor risk through the latest evidence of comprehensive observational studies. We tried to explore the dose-response relationship between vitamins and brain tumors, hoping to provide evidence for preventing brain tumors.

## Methods

### Search strategy

A comprehensive search was conducted for available articles published in English using databases such as the Cochrane Library, PubMed, Web of Science, and Embase up to 19 December 2021. The Cochrane Library search terms used for the title, abstract, and keywords were (“glioma” OR “brain cancer” OR “brain tumor”) combined with (“diet” OR “food” OR “lifestyle” OR “nutrition” OR “nutrient” OR “vitamin” OR “carotenoid” OR “carotene” OR “ascorbic acid” OR “thiamine” OR “riboflavin” OR “tocopherol” OR “25 hydroxyvitamin D” OR “folic acid” OR “nicotinic acid” OR “antioxidant”). The same retrieval strategy was also applied to the other databases. No document type or other relevant restrictions were used in the retrieval process, and unpublished articles were excluded. Two investigators independently searched articles and reviewed all retrieved studies. A third author settled any disagreements between the two authors. In addition, we explored the references of published meta-analyses to identify other potential articles.

### Inclusion and exclusion criteria

The following inclusion criteria were used: (1) the studies were using a cohort design or a case-control design; (2) the exposure of interest was vitamin intake or serum vitamin concentration; and (3) the ending outcome was brain tumors.

The exclusion criteria of the meta-analysis were as follows: (1) non-observational study (reviews, case reports, and clinical trials); (2) lack of effect size and 95%CI which were available for the highest category of vitamin vs. lowest category of vitamin; and (3) If multiple studies used data from the same population, the study with the largest sample size was included in this meta-analysis.

### Data extraction

Two investigators extracted the following information from the included study independently: the first author, year of publication, country, study population, study type, age, sex, sample size, number of cases, disease, vitamin source, vitamin type, vitamin level, effect size, and 95% CI extracted from the most adjusted model. If there was disagreement between the two authors about the appropriateness of the data, it was resolved by consensus with a third author.

### Quality assessment

Two investigators evaluated each study and handed it over to a third party for adjudication in case of disagreement. Since the included articles were observational studies, the Newcastle-Ottawa scale (NOS) was used to evaluate the quality of the study and the possible risk of bias (18).

### Statistical analysis

Stata 14.0 software was used for data analysis. We pooled effect size estimations by combining the multivariable-adjusted effect size and 95%CI of the highest compared with the lowest vitamins. *I*^2^ statistics assessed heterogeneity between the studies. Suppose the heterogeneity was not statistically significant (*I*^2^ <50% and *P* > 0.10), the fixed-effects model was used to pool them. Otherwise, the random-effects model was used. We conducted a subgroup analysis to determine whether the heterogeneity of the study came from disease (brain tumor and glioma), vitamin source (diet and supplements), study population (pregnancy exposure and self-exposure), study type (case-control study and cohort study), and study quality (>7 points and ≤ 7 points), to explore the potential sources of heterogeneity. We used sensitivity analysis to assess each study's relative impact on the total effect size by successively omitting one study when determining the effect size. For publication bias, Egger's test and Begg's test were used to detect it.

Subsequently, we also explored the dose-response relationship between vitamins and brain tumor risk. The method developed by Greenland and Longnecker was used to analyze the dose-response relationship in this study (19). For this method, we needed to extract at least three groups of vitamin intakes or serum vitamin concentration, number of participants, number of cases, effect size, and 95% CI in each study. The median or average vitamin corresponding to each group was used for risk estimation for each study. Suppose the median or average vitamin of each group was not provided, the midpoint of each group's upper and lower limits should be designated as the intermediate exposure level. If the highest group was open, we assumed that the interval width was the same as the second-highest category. *Q*-value was applied to assess between-study heterogeneity. Unless otherwise noted, two-tailed *P* < 0.05 was accepted as statistically significant.

## Results

### Study characteristics

**Figure 1:**
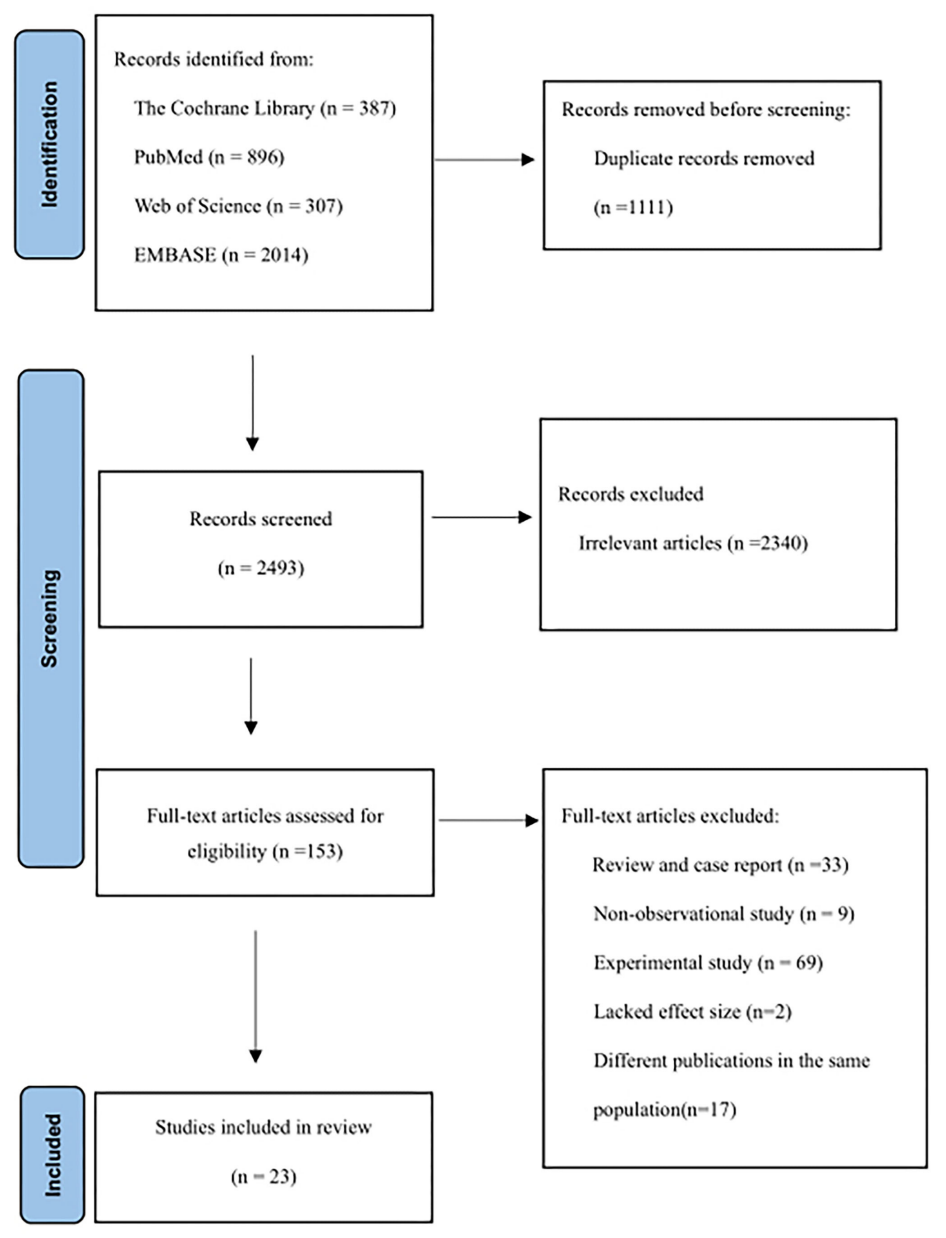
Flow diagram outlining the systematic search and articles selection process.

Figure 1 shows the articles screening process of this study. A total of 3,604 articles were retrieved, including 387 from the Cochran Library, 896 from PubMed, 307 from Web of Science, and 2014 from Embase. After excluding duplicates between different databases, titles and abstracts of 2,493 articles were reviewed. A total of 2,340 articles were excluded because they were not related to the aim of the study. Then, 153 articles were reviewed in full text, and 130 articles were excluded due to non-observational studies, animal/cell experiments, reviews, lacked effect size, or duplication of the study population. A total of 23 articles were included (11, 12, 14–17, 20–36).

Table 1 summarizes the 23 articles and characteristics included in this meta-analysis. All studies included 1,347,426 controls and 6,449 patients with brain tumors. Among them, the patients in eight studies were minors, and the participants in the other studies were 18–80 years old. The included studies were mainly concentrated in North America (America and Canada) and Europe (Britain, Germany, and Sweden). A few studies were completed by Australia, China, and Iran, including 20 case-control studies and 4 cohort studies. These studies provided brain tumor-related results for 6 vitamin intakes: vitamin A, vitamin B, vitamin C, vitamin E, β-carotene, and folate. In addition, there were the results of serum 25-hydroxyvitamin D and serum α-tocopherol. Around 50% of the studies had a NOS score of eight or above.

**Table 1:** Characteristics of studies investigating the association between vitamins and brain tumors.

**Study**	**Year**	**Country**	**Study type**	**Age**	**Sex** ^a^	**Population** ^b^	**Sample size**	**Case**	**Disease**	**Source**	**Vitamin**	**Effect size (95%CI)**	**Quality score**
Howe et al. (20)	1989	Canada	Case-control	<19	Both	Self-exposure	146	52	Brain tumor	Supplement	Vitamin C	0.91(0.43–1.93)	8
Boeing et al. (21)	1993	Germany	Case-control	25–75	Both	Self-exposure	470	93	Glioma	Diet	Vitamin C	0.90(0.50–1.70)	7
Bunin et al. (22)	1994	America	Case-control	0–6	Female	Pregnancy exposure	288	144	Glioma	Diet	Vitamin A	0.70(0.30–1.40)	8
											Vitamin C	0.70(0.40–1.50)	
											Vitamin E	0.70(0.30–130)	
											β-carotene	1.00(0.50–2.00)	
											Folate	1.00(0.50–2.10)	
Gile et al. (12)	1994	Australia	Case-control	20–70	Both	Self-exposure	818	409	Glioma	Diet	Vitamin A	1.64(1.13–2.37)	7
											Vitamin C	0.96(0.42–2.15)	
											Vitamin E	1.42(1.00–2.02)	
											β-carotene	0.85(0.59–1.23)	
Blowers et al. (23)	1997	America	Case-control	25–74	Female	Self-exposure	188	94	Glioma	Diet	Vitamin A	0.70(0.30–1.90)	7
											Vitamin C	1.50(0.60–4.10)	
											Vitamin E	2.20(0.80–5.70)	
Lee et al. (24)	1997	America	Case-control	>20	Both	Self-exposure	857	419	Glioma	Supplement	Vitamin C	0.76(0.56–1.01)	8
											Vitamin E	0.79(0.55–1.14)	
Preston-Martin et al. (25)	1998	Britain	Case-control	0–19	Female	Pregnancy exposure	952	373	Brain tumor	Supplement	Vitamin A	0.40(0.20–0.80)	7
											Vitamin C	0.50(0.30–0.90)	
											Vitamin E	0.50(0.30–0.80)	
											Folate	0.50(0.30–0.80)	
Hu et al. (26)	1999	China	Case-control	20–74	Both	Self-exposure	287	129	Brain tumor	Diet	Vitamin C	0.78(0.20–4.10)	6
											Vitamin E	0.16(0.10–0.50)	
											β-carotene	0.38(0.10–1.60)	
Schwartzbaum et al. (16)	2000	America	Case-control	36–69	Both	Self-exposure	69	34	Glioma	Serum	α-tocopherol	0.36(0.10–1.10)	8
Chen et al. (11)	2002	America	Case-control	≥21	Both	Self-exposure	685	236	Glioma	Diet	Vitamin A	0.50(0.30–0.80)	7
											Vitamin C	0.90(0.50–1.50)	
											Vitamin E	0.80(0.50–1.40)	
											β-carotene	0.50(0.30–0.90)	
											Folate	0.90(0.50–1.50)	
Tedeschi-Blok et al. (14)	2006	America	Case-control	≥20	Both	Self-exposure	1,648	802	Glioma	Diet	Vitamin C	0.70(0.51–0.94)	9
											Vitamin E	0.91(0.62–1.34)	
											β-carotene	0.72(0.54-0.98)	
Holick et al. (27)	2007	America	Cohort	25–75	Both	Self-exposure	2,29,637	296	Glioma	Diet	β-carotene	0.92(0.64–1.32)	8
Michaud et al. (28)	2009	America	Cohort	25–75	Both	Self-exposure	2,19,334	335	Glioma	Diet	Vitamin C	0.88(0.62–1.26)	8
											Vitamin E	0.98(0.67–1.43)	
Dubrow et al. (15)	2010	America	Cohort	50–71	Both	Self-exposure	5,45,770	585	Glioma	Diet	Vitamin C	1.26(0.96–1.66)	7
											Vitamin E	1.17(0.90–1.53)	
Stalberg et al. (29)	2010	Sweden	Case-control	0–15	Female	Pregnancy exposure	1,037	512	Brain tumor	Supplement	Folate	0.60(0.30–1.10)	9
Miline et al. (17)	2012	Australia	Case-control	0–14	Female	Pregnancy exposure	1,014	287	Brain tumor	Supplement	Vitamin A	1.17(0.72–1.90)	8
											Vitamin B	1.03(0.68–1.56)	
											Vitamin C	0.96(0.64–1.46)	
											Folate	0.60(0.38–0.98)	
Greenop et al. (30)	2014	Australia	Case-control	0-15	Female	Pregnancy exposure	1,019	293	Brain tumor	Diet	Vitamin B	1.04(0.72-1.50)	8
											Folate	0.70(0.48–1.02)	
Bhatti et al. (31)	2015	America	Case-control	0–15	Both	Self-exposure	494	247	Brain tumor	Serum	25-hydroxyvitamin D	1.30(0.80–2.20)	8
Greenop et al. (32)	2015	Australia	Case-control	3–15	Both	Self-exposure	739	216	Brain tumor	Diet	Vitamin B	1.23(0.80–1.89)	6
											Folate	0.63(0.41–0.97)	
Zigmont et al. (33)	2015	America	Case-control	20–65	Both	Self-exposure	1,704	592	Glioma	Serum	25-hydroxyvitamin D	1.04(0.73–1.47)	7
Huang et al. (34)	2017	America	Case-control	50–69	Male	Self-exposure	128	64	Glioma	Serum	α-tocopherol	0.65(0.44–0.96)	8
Heydari et al. (35)	2020	Iran	Case-control	20–75	Both	Self-exposure	384	128	Glioma	Diet	Vitamin B	0.35(0.13–0.97)	7
											Vitamin C	0.14(0.05–0.36)	
											Vitamin E	0.83(0.35–1.97)	
											β-carotene	0.99(0.45–2.18)	
Yue et al. (36)	2021	America	Cohort	40–69	Both	Self-exposure	3,46,812	444	Glioma	Serum	25-hydroxyvitamin D	0.87(0.68–1.11)	8
Yue et al. (36)	2021	America	Case-control	30–55	Both	Self-exposure	252	84	Glioma	Serum	25-hydroxyvitamin D	0.97(0.51–1.85)	8
											α-tocopherol	0.61(0.29–1.32)	

^a^Sex of exposed population.

^b^The population was divided into pregnancy exposure and self-exposure.

### Effect size estimations of risk for the association between vitamins and brain tumor

The effect size estimations between all vitamins and risk of brain tumors are shown in Table 2. For vitamin intake, compared with the lowest intakes, the highest intakes of vitamin C (RR = 0.81, 95%CI:0.66–0.99, *I*^2^ = 54.7%, *P*_*for heterogeneity*_ = 0.007), β-carotene (RR = 0.78, 95%CI:0.66–0.93, *I*^2^ = 0, *P*_*for heterogeneity*_ = 0.460), and folate (RR = 0.66, 95%CI:0.55–0.80, *I*^2^ = 0, *P*_*for heterogeneity*_ = 0.661) significantly reduced the risk of brain tumor, while the highest intakes of vitamin A (RR = 0.79, 95%CI:0.48–1.29, *I*^2^ = 77.9%, *P*_*for heterogeneity*_ < 0.001), vitamin B (RR = 1.03, 95%CI:0.82–1.29, *I*^2^ = 41.1%, *P*_*for heterogeneity*_ = 0.165), and vitamin E (RR = 0.83, 95%CI:0.63–1.10, *I*^2^ = 73.5%, *P*_*for heterogeneity*_ < 0.001) were not related to the incidence of brain tumor. For serum vitamin, compared with the lowest concentrations, the highest concentrations of serum α-tocopherol (RR = 0.61, 95%CI:0.44–0.86, *I*^2^ = 0, *P*_*for heterogeneity*_ = 0.656), while the highest concentrations of serum 25-hydroxyvitamin D (RR = 0.97, 95%CI:0.81–1.16, *I*^2^ = 0, *P*_*for heterogeneity*_ = 0.533) was not related to the incidence of brain tumor (Supplementary Figures 1–8).

**Table 2:** A meta-analysis of the association between vitamins and brain tumors.

**Vitamins**	**Number of studies**	**RR (95%CI)**	***I**^2^* **(%)**	***P***_for heterogeneity_
**Intake**				
Vitamin A	6	0.79(0.48–1.29)	77.9%	<0.001
Vitamin B	4	1.03(0.82–1.29)	41.1%	0.165
Vitamin C	14	0.81(0.66–0.99)	54.7%	0.007
Vitamin E	11	0.83(0.63–1.10)	73.5%	<0.001
β-carotene	7	0.78(0.66–0.93)	0	0.460
Folate	7	0.66(0.55–0.80)	0	0.661
**Serum**				
Serum 25-hydroxyvitamin D	4	0.97(0.81–1.16)	0	0.533
Serum α-tocopherol	3	0.61(0.44–0.86)	0	0.656

### Subgroup analysis

For disease, vitamin E was statistically significant in the brain tumor subgroup (RR = 0.30, 95%CI:0.10–0.90). For vitamin source, vitamin C was statistically significant in the supplement subgroup (RR = 0.77, 95%CI:0.60–0.98). For the study population, vitamin E was statistically significant in the pregnancy exposure subgroup (RR = 0.55, 95%CI:0.37–0.83). For the study area, vitamin A was statistically significant in the subgroups of America, Europe, and Australia (America: RR = 0.57, 95%CI:0.39–0.84; Europe: RR = 0.40, 95%CI:0.20–0.80; and Australia: RR = 1.44, 95%CI:1.04–1.99), and the heterogeneity of vitamin A decreased from 77.9 to 15.0%. For study type, vitamin C was statistically significant in the case-control study subgroup (RR = 0.75, 95%CI:0.61–0.93) (Table 3).

**Table 3:** Subgroup analysis for the association between vitamins and brain tumors.

**Vitamin**	**Subgroup**	**Number**	**RR (95%CI)**	***I**^2^* **(%)**	***P**_*for heterogeneity*_*
**Vitamin A**	**Disease**				
	Glioma	4	0.82(0.42–1.61)	81.1	0.001
	Brain tumor	2	0.70(0.25–2.01)	83.8	0.013
	**Vitamin source**				
	Diet	4	0.82(0.42–1.61)	81.1	0.001
	Supplement	2	0.70(0.25–2.01)	83.8	0.013
	**Study population**				
	Pregnancy exposure	3	0.71(0.37–1.38)	68.3	0.043
	Self-exposure	3	0.85(0.36–2.03)	86.7	0.001
	**Study area**				
	America	3	0.57(0.39–0.84)	0	0.692
	Europe	1	0.40(0.20–0.80)	-	-
	Australia	2	1.44(1.04–1.99)	15.0	0.278
	**Study quality**				
	≤ 7	4	0.71(0.33–1.52)	85.8	<0.001
	>7	2	0.99(0.62–1.59)	18.3	0.269
**Vitamin B**	**Study population**				
	Pregnancy exposure	2	1.04(0.79–1.36)	0	0.973
	Self-exposure	2	1.01(0.68–1.50)	80.3	0.024
	**Study quality**				
	≤ 7	2	1.01(0.68–1.50)	80.3	0.024
	>7	2	1.04(0.79–1.36)	0	0.973
**Vitamin C**	**Disease**				
	Glioma	10	0.82(0.64–1.05)	63.5	0.003
	Brain tumor	4	0.77(0.54–1.09)	17.9	0.301
	**Vitamin source**				
	Diet	10	0.82(0.62–1.09)	62.1	0.005
	Supplement	4	0.77(0.60–0.98)	18.3	0.299
	**Study population**				
	Pregnancy exposure	3	0.72(0.48–1.08)	43.0	0.173
	Self-exposure	11	0.84(0.66–1.07)	58.8	0.007
	**Study area**				
	America	8	0.89(0.73–1.08)	38.1	0.126
	Europe	2	0.66(0.37–1.17)	49.1	0.161
	Australia	2	0.96(0.66–1.39)	0	1.000
	Asia	2	0.30(0.06–1.59)	71.3	0.062
	**Study type**				
	Case-control	12	0.75(0.61–0.93)	39.5	0.077
	Cohort	2	1.07(0.76–1.52)	59.4	0.116
	**Study quality**				
	≤ 7	8	0.77(0.50-1.19)	71.8	0.001
	>7	6	0.79(0.68-0.93)	0	0.829
**Vitamin E**	**Disease**				
	Glioma	9	1.02(0.85–1.21)	28.0	0.195
	Brain tumor	2	0.30(0.10–0.90)	82.2	0.018
	**Vitamin source**				
	Diet	9	0.89(0.65–1.23)	73.0	<0.001
	Supplement	2	0.65(0.42–1.01)	53.6	0.142
	**Study population**				
	Pregnancy exposure	2	0.55(0.37–0.83)	0	0.455
	Self-exposure	9	0.90(0.67–1.21)	73.6	<0.001
	**Study area**				
	America	7	0.97(0.81–1.15)	16.8	0.302
	Europe	1	0.50(0.31–0.82)	-	-
	Australia	1	1.42(1.00–2.02)	-	-
	Asia	2	0.36(0.07–1.81)	86.6	0.006
	**Study type**				
	Case-control	9	0.77(0.53–1.10)	76.1	<0.001
	Cohort	2	1.10(0.89–1.37)	0	0.453
	**Study quality**				
	≤ 7	7	0.80(0.50–1.29)	83.4	<0.001
	>7	4	0.87(0.71–1.07)	0	0.792
**β-carotene**	**Study quality**				
	≤ 7	4	0.73(0.55–0.97)	23.2	0.272
	>7	3	0.81(0.65–1.01)	0	0.489
**Folate**	**Disease**				
	Glioma	2	0.94(0.60–1.45)	0	0.819
	Brain tumor	5	0.62(0.50–0.76)	0	0.884
	**Vitamin source**				
	Diet	5	0.71(0.57–0.88)	0	0.667
	Supplement	2	0.53(0.36–0.79)	0	0.661
	**Study population**				
	Pregnancy exposure	5	0.64(0.51–0.80)	0	0.597
	Self-exposure	2	0.72(0.51–1.01)	0.3	0.317
	**Study quality**				
	≤ 7	3	0.64(0.48–0.85)	18.6	0.293
	>7	4	0.68(0.53–0.88)	0	0.673

For vitamin C, when Heydari's study (35) was excluded, the results of all studies and brain tumor risk remained significant, but the heterogeneity decreased significantly (RR = 0.87, 95%CI:0.77–0.99, *I*^2^ = 23.4%, *P*_*for heterogeneity*_ = 0.207). Similarly, excluding another study (26), the heterogeneity of vitamin E was also significantly reduced (RR = 0.94, 95%CI:0.76–1.16, *I*^2^ = 52.3%, *P*_*for heterogeneity*_ = 0.026). It was speculated that these studies might be the main reasons for the heterogeneity of vitamin C and brain tumor risk. The sources of heterogeneity between vitamin B intake and brain tumor effect size estimations were unclear.

The heterogeneity of β-carotene, folate, serum 25-hydroxyvitamin D, and serum α-tocopherol was minimal, so no subgroup analysis was carried out.

### Sensitivity analysis and publication bias

The sensitivity analysis showed that no individual study had an excessive influence on the association of vitamins and brain tumors when we removed one individual study at a time. This suggested the results of this meta-analysis were relatively stable (Table 4).

**Table 4:** Sensitivity analysis and publication bias.

	**Influential analysis**	**Egger's test**	**Begg's test**
Vitamin A	0.38–1.48	0.169	0.707
Vitamin B	0.74–1.37	0.101	0.734
Vitamin C	0.63–1.03	0.296	0.743
Vitamin E	0.58–1.17	0.170	0.276
β-carotene	0.61–1.00	0.658	0.764
Folate	0.52–0.85	0.462	0.764
Serum 25-hydroxyvitamin D	0.76–1.42	0.300	0.734
Serum α-tocopherol	0.28–1.00	0.302	0.296

Publication bias was evaluated by Egger's regression test and Begg's rank correlation method. The *P*-value of publication bias of vitamins was more significant than 0.1, suggesting that the difference was not statistically significant, thus there was no publication bias (Table 4).

### Dose-response relationship

**Figure 2:**
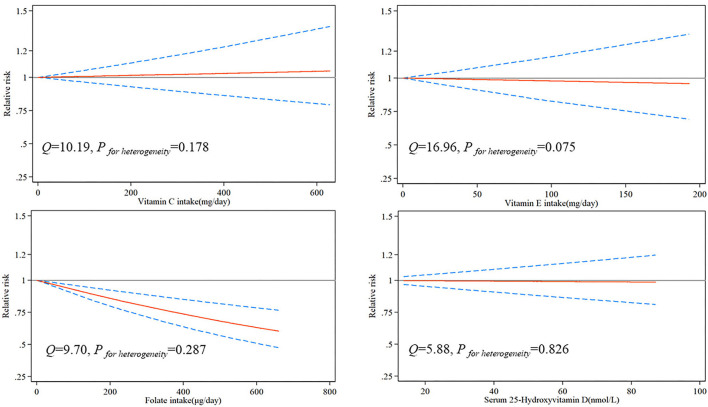
Risk between vitamins and brain tumors estimates from dose-response meta-analysis.

Due to the limited number of available articles, only vitamin C, vitamin E, folate, and serum 25-hydroxyvitamin D could be analyzed for dose-response relationship from nine articles. The dose-response relationship between vitamins and the risk of brain tumor is shown in Figure 2. There was a significant linear dose-response relationship between folate and brain tumor, and increasing 100 μg folate per day reduced brain tumor risk by 7% (*P*_−*nonlinearity*_ = 0.534, 95%CI:0.90–0.96). Although vitamin C, vitamin E, and serum 25-hydroxyvitamin D had similar linear trends, the results were insignificant due to insufficient studies.

## Discussion

Based on 23 articles on vitamins and brain tumors published from 1989 to 2021, a total of 1,347,426 controls and 6,449 patients with brain tumors were included. Our meta-analysis results showed that for vitamin intake, higher intakes of vitamin C, β-carotene, and folate had a significant protective effect on brain tumors. For vitamin concentration *in vivo*, high serum α-tocopherol concentration could significantly reduce the risk of brain tumors. There was no significant correlation between vitamin A, vitamin B, vitamin E, and serum 25-hydroxyvitamin D and the incidence of brain tumors. There was a significant linear dose-response relationship between folate and brain tumor, and increasing per 100 μg/day folate intake reduced brain tumor risk by 7%. Although there were similar linear trends between vitamin C, vitamin E, serum 25-hydroxyvitamin D, and brain tumor risk, the results were insignificant. This might be due to the limited number of articles that analyzed the dose-response relationship between these vitamins and brain tumors.

We explored the sources of heterogeneity through disease conditions, vitamin sources, study population, study area, study type, and study quality. The results of vitamin A were not significant, but through subgroup analysis, it was found that its heterogeneity mainly comes from the study area. The results of European and American studies showed that vitamin A had a protective effect on brain tumors. In contrast, the results of Australian studies suggested that excessive intake of vitamin A could significantly increase the risk of brain tumors. It was speculated that there was a significant difference in the intake of vitamin A due to different eating habits in the northern and southern hemispheres. Heydari's study contributed the most heterogeneity to the meta-analysis of vitamin C and brain tumors (35). It is well-known that the primary dietary sources of vitamin C are vegetables and fruits. Heydari's research showed that about 60% of Iranian adults had low fruit and vegetable intake, and the average intake of vitamin C in the control population was 143 mg/day (35), while half of the American people in Michaud et al.'s (28) study had more than 232 mg/day. Therefore, there were significant differences in vitamin C intake between the Iranian and other populations, resulting in considerable heterogeneity in this study. In addition, in the study of Hu et al. (26) only 57 kinds of food were investigated. In comparison, most of the food types investigated were more than 80 kinds in other studies, which might not obtain accurate vitamin intake, resulting in the heterogeneity of vitamin E.

Compared with other tissues, the brain has active metabolism and can produce many reactive oxygen species. Still, the brain has low antioxidant defense ability, leading to DNA loss and tumor development (37). A case-control study of dietary antioxidants and glioma conducted by Tedeschi Blok et al. found that a higher intake of vitamin C was associated with a reduced risk of glioma (RR = 0.70, 95% CI:0.51–0.94) (14). Preston-martin et al. found that prenatal vitamin C supplementation could significantly reduce the risk of brain tumors in children (RR = 0.50, 95% CI:0.30–0.90). There was a dose-response relationship between intake and brain tumor risk (25). On the one hand, vitamin C could inhibit and reduce N-acetyltransferase activity and the formation of 2-aminofluorene-DNA adduct in rat C6 glioma cells in a dose-dependent manner (38). On the other hand, the rat experiment found that two markers related to brain tumor proliferation, platelet-derived growth factor receptor (PDGFRb), were found in rats fed with antioxidants such as vitamin C. Furthermore, Ki-67 transcripts were significantly reduced, suggesting that vitamin C could limit the invasiveness of brain tumors (39). In addition, some studies had found that vitamin C could inhibit the growth of glioblastoma through the caspase-3 death pathway and then assist the treatment of glioblastoma with methotrexate (40). Although our results did not find the protective effect of vitamin E intake on brain tumors, which was consistent with the results of two cohort studies in the United States (15, 28), the survival rate of patients with high vitamin E intake was higher in patients with grade III malignant glioma (41). Moreover, vitamin E derivatives reduced the incidence of pituitary tumors in X-ray-irradiated mice (42). We could not rule out the individual metabolic differences of vitamin E, resulting in inconsistent results. The results of prospective glioma serum metabolomics showed that serum α-tocopherol (the most bioactive form of vitamin E) concentrations were significantly negatively correlated with glioma risk (34), which was consistent with the results of the meta-analysis of serum α-tocopherol. We found that vitamin A intake had no significant effect on brain tumors. At present, there was no cohort study to explore the association between vitamin A and brain tumors, and the conclusions of case-control studies were inconsistent. Still, the previous meta-analysis showed that vitamin A could reduce the risk of glioma (RR = 0.80, 95% CI = 0.62–0.98, *I*^2^ = 54.9%) (13). There were few studies on the mechanism of vitamin A and brain tumors. Some studies believed that brain tumors were closely related to retinoic acid, the metabolite of vitamin A and the level of retinoic acid-binding protein 2 in brain tumors were low related to the survival rate of patients (43). Although the relationship between vitamin A and brain tumors was not clear, β-carotene, as a precursor of vitamin A, showed a protective effect on brain tumors. Tedeschi Blok et al. found that the average intake of β-carotene in the control population was 252.8 mg/day (RR = 0.72, 95% CI:0.54–0.98) higher than that in patients with brain tumors, and the serum β-carotene concentration in patients with brain tumors was also significantly lower than that in healthy people (44). Cell experiments confirmed that β-carotene could effectively inhibit DNA synthesis in growing C-6 glioma cells (45). In addition, in the study of vegetable intake and brain tumors, it was also found that compared with other vegetables, orange vegetables rich in β-carotene had a stronger protective effect on brain tumors (11, 46). This study was the first time that folate could reduce the risk of brain tumors in the meta-analysis, which was consistent with the results of many epidemiological studies (17, 32). In recent years, the effect of folate on brain tumors had attracted much attention, especially in children. It had been found that the deficiency of folate metabolism might play an important role in the pathogenesis of some specific subtypes of brain tumors in children, especially embryonic central nervous system tumors (47). The mechanism might be related to the folate receptor. On the one hand, the folate receptor was found to be overexpressed in ependymoma, medulloblastoma, and other common malignant tumors of children's central nervous system (48, 49). Moreover, folate supplementation can enhance DNA remethylation through SP1/SP3 mediated transcriptional upregulation of DNMT3a and DNMT3b protein-coding genes to limit the invasiveness of glioma (50). In addition, targeted folate metabolism had selective cytotoxicity to glioma stem cells and can effectively cooperate with differentiation therapy to eliminate tumor-initiating cells in xenogeneic glioma grafts (51). However, only a few studies had reported the association between vitamin B and brain tumors, and the results were not significant. We also did not find any relevant research on dietary vitamin D and brain tumors. Since sunlight could promote vitamin D synthesis *in vivo*, it seemed more scientific to evaluate its effect on brain tumors through vitamin D concentration *in vivo*. Although experimental studies had shown that Vitamin D could promote cell cycle arrest and induce cell death to suppress tumor growth in glioblastoma (52, 53). However, no significant effect of vitamin D on brain tumors was found in epidemiological studies (31, 33).

So far, this was the largest meta-analysis of vitamins and brain tumors. Therefore, this study had several advantages. First, this study was the first meta-analysis involving the effects of multivitamins on brain tumors, including seven vitamins. The protective effects of β-carotene and folate on brain tumors were found in a meta-analysis for the first time. The dose-response relationship between folate and the risk of brain tumors was explored, which provided new evidence for preventing brain tumors. Second, this study also explored the relationship between vitamin concentration in serum and brain tumors to confirm the actual effect of vitamin intake. Third, we thoroughly discussed the sources of heterogeneity of the research results and improved the accuracy of the significant results. However, the study also had limitations. This study failed to further explore the relationship between vitamin and brain tumor subtypes. The incidence rate of brain tumors is very low, and the annual incidence rate was only 22.6/10 million (1). Although our current study included most observational studies of vitamins and brain tumors, the sample size was still limited compared with other tumor studies. In addition, glioma is the most common brain tumor. Therefore, most of the current related studies focused on gliomas or brain tumors, especially in meta-analyses and systematic reviews (54, 55). In the search process, we did not find any studies that met the inclusion criteria, and the subjects had meningioma, germ cell tumor, or other brain tumor diseases. As a considerable part of the exposed population was pregnant women, and the outcomes of relevant studies were child brain tumors, this might cause some heterogeneity in the analysis process. However, we discussed the results of pregnancy exposure and self-exposure in the subgroup analysis and obtained similar results in some vitamins (such as folate). Most studies could only provide the source of intake of a particular vitamin (diet or supplement), so it was impossible to comprehensively evaluate the relationship between the overall intake of vitamins and brain tumors. Next, for the study of vitamin concentrations *in vivo*, only vitamin D and vitamin E provide sufficient articles, and there were too few studies on the concentrations of other vitamins to explore their correlation fully. We hope to improve the relevant analysis by adding more articles in future research.

## Conclusion

In summary, the current meta-analysis shows that higher intakes of vitamin C, β-carotene, and folate can reduce the risk of brain tumors. At the same time, high serum α-tocopherol concentration also has a protective effect on brain tumors. Therefore, vitamins may provide new ideas for the prevention of brain tumors. In the future, we should pay attention to the compounds with antioxidant effects in the diet to further discover their effects on brain tumors.

## Data availability statement

The original contributions presented in the study are included in the article/Supplementary material, further inquiries can be directed to the corresponding author/s.

## Author contributions

WL and WZ contributed to the conception or design of the work, WZ, JJ, and YH contributed to searching the databases. WZ, JJ, and XL contributed to the acquisition, analysis, or interpretation of data for the work. WZ, XL, and SY proofread and modified the language. WL and FC reviewed and edited the manuscript. All authors have read and approved the final manuscript.

## Funding

This study was supported by the National Natural Science Foundation of Beijing (No. J200003) and the National Science and Technology Major Project of China (No. 2016ZX09101017).

## Conflict of interest

The authors declare that the research was conducted in the absence of any commercial or financial relationships that could be construed as a potential conflict of interest.

## Publisher's note

All claims expressed in this article are solely those of the authors and do not necessarily represent those of their affiliated organizations, or those of the publisher, the editors and the reviewers. Any product that may be evaluated in this article, or claim that may be made by its manufacturer, is not guaranteed or endorsed by the publisher.

## References

[1] Barnholtz-Sloan JS Ostrom QT Cote D. Epidemiology of brain tumors. Neurol Clin. (2018) 36:395–419. 10.1016/j.ncl.2018.04.001 30072062

[2] McNeill KA. Epidemiology of brain tumors. Neurol Clin. (2016) 34:981–98. 10.1016/j.ncl.2016.06.014 27720005

[3] Butowski NA. Epidemiology and diagnosis of brain tumors. Continuum. (2015) 21:301–13. 10.1212/01.CON.0000464171.50638.fa 25837897

[4] D'Archivio M Santangelo C Scazzocchio B Vari R Filesi C Masella R . Modulatory effects of polyphenols on apoptosis induction: Relevance for cancer prevention. Int J Mol Sci. (2008) 9:213–28. 10.3390/ijms9030213 19325744 PMC2635670

[5] Pouliquen D Olivier C Hervouet E Pedelaborde F Debien E Le Cabellec MT . Dietary prevention of malignant glioma aggressiveness, implications in oxidant stress and apoptosis. Int J Cancer. (2008) 123:288–95. 10.1002/ijc.23513 18412241

[6] Khoshyomn S Nathan D Manske GC Osler TM Penar PL. Synergistic effect of genistein and BCNU on growth inhibition and cytotoxicity of glioblastoma cells. J Neurooncol. (2002) 57:193–200. 10.1023/a:1015765616484 12125982

[7] Pawlowska E Szczepanska J Blasiak J. Pro- and antioxidant effects of vitamin c in cancer in correspondence to its dietary and pharmacological concentrations. Oxid Med Cell Longev. (2019) 2019:7286737. 10.1155/2019/7286737 31934267 PMC6942884

[8] Abraham A Kattoor AJ Saldeen T Mehta JL. Vitamin E and its anticancer effects. Crit Rev Food Sci Nutr. (2019) 59:2831–8. 10.1080/10408398.2018.1474169 29746786

[9] Bielecka J Markiewicz-Zukowska R. The influence of nutritional and lifestyle factors on glioma incidence. Nutrients. (2020) 12:1812. 10.3390/nu12061812 32560519 PMC7353193

[10] Jeon SM Shin EA. Exploring vitamin D metabolism and function in cancer. Exp Mol Med. (2018) 50:1–14. 10.1038/s12276-018-0038-9 29657326 PMC5938036

[11] Chen H Ward MH Tucker KL Graubard BI McComb RD Potischman NA . Diet and risk of adult glioma in eastern Nebraska, United States. Cancer Causes Control. (2002) 13:647–55. 10.1023/a:1019527225197 12296512

[12] Giles GG McNeil JJ Donnan G Webley C Staples MP Ireland PD . Dietary factors and the risk of glioma in adults: results of a case-control study in Melbourne, Australia. Int J Cancer. (1994) 59:357–62. 10.1002/ijc.2910590311 7927941

[13] Lv W Zhong X Xu L Han W. Association between dietary Vitamin a intake and the risk of Glioma: evidence from a meta-analysis. Nutrients. (2015) 7:8897–904. 10.3390/nu7115438 26516909 PMC4663566

[14] Tedeschi-Blok N Lee M Sison JD Miike R Wrensch M. Inverse association of antioxidant and phytoestrogen nutrient intake with adult glioma in the San Francisco Bay area: a case-control study. BMC Cancer. (2006) 6:148. 10.1186/1471-2407-6-148 16749939 PMC1513391

[15] Dubrow R Darefsky AS Park Y Mayne ST Moore SC Kilfoy B . Dietary components related to N-nitroso compound formation: a prospective study of adult glioma. Cancer Epidemiol Biomarkers Prev. (2010) 19:1709–22. 10.1158/1055-9965.EPI-10-0225 20570910 PMC2901412

[16] Schwartzbaum JA Cornwell DG. Oxidant stress and glioblastoma multiforme risk: serum antioxidants, gamma-glutamyl transpeptidase, and ferritin. Nutr Cancer. (2000) 38:40–9. 10.1207/S15327914NC381_7 11341043

[17] Milne E Greenop KR Bower C Miller M van Bockxmeer FM Scott RJ . Maternal use of folic acid and other supplements and risk of childhood brain tumors. Cancer Epidemiol Biomarkers Prev. (2012) 21:1933–41. 10.1158/1055-9965.EPI-12-0803 22941336

[18] Stang A. Critical evaluation of the Newcastle-Ottawa scale for the assessment of the quality of nonrandomized studies in meta-analyses. Eur J Epidemiol. (2010) 25:603–5. 10.1007/s10654-010-9491-z 20652370

[19] Greenland S Longnecker MP. Methods for trend estimation from summarized dose-response data, with applications to meta-analysis. Am J Epidemiol. (1992) 135:1301–9. 10.1093/oxfordjournals.aje.a116237 1626547

[20] Howe GR Burch JD Chiarelli AM Risch HA Choi BC. An exploratory case-control study of brain tumors in children. Cancer Res. (1989) 49:4349–52. 2743324

[21] Boeing H Schlehofer B Blettner M Wahrendorf J. Dietary carcinogens and the risk for glioma and meningioma in Germany. Int J Cancer. (1993) 53:561–5. 10.1002/ijc.2910530406 8436429

[22] Bunin GR Kuijten RR Boesel CP Buckley JD Meadows AT. Maternal diet and risk of astrocytic glioma in children: a report from the childrens cancer group (United States and Canada). Cancer Causes Control. (1994) 5:177–87. 10.1007/BF01830264 8167265

[23] Blowers L Preston-Martin S Mack WJ. Dietary and other lifestyle factors of women with brain gliomas in Los Angeles County (California, USA). Cancer Causes Control. (1997) 8:5–12. 10.1023/a:1018437031987 9051317

[24] Lee M Wrensch M Miike R. Dietary and tobacco risk factors for adult onset glioma in the San Francisco Bay Area (California, USA). Cancer Causes Control. (1997) 8:13–24. 10.1023/a:1018470802969 9051318

[25] Preston-Martin S Pogoda JM Mueller BA Lubin F Holly EA Filippini G . Prenatal vitamin supplementation and risk of childhood brain tumors. Int J Cancer Suppl. (1998) 11:17–22. 9876471

[26] Hu J La Vecchia C Negri E Chatenoud L Bosetti C Jia X . Diet and brain cancer in adults: a case-control study in northeast China. Int J Cancer. (1999) 81:20–3. 10.1002/(sici)1097-0215(19990331)81:1<20::aid-ijc4>3.0.co;2-2 10077146

[27] Holick CN Giovannucci EL Rosner B Stampfer MJ Michaud DS. Prospective study of intake of fruit, vegetables, and carotenoids and the risk of adult glioma. Am J Clin Nutr. (2007) 85:877–86. 10.1093/ajcn/85.3.877 17344512

[28] Michaud DS Holick CN Batchelor TT Giovannucci E Hunter DJ. Prospective study of meat intake and dietary nitrates, nitrites, and nitrosamines and risk of adult glioma. Am J Clin Nutr. (2009) 90:570–7. 10.3945/ajcn.2008.27199 19587083 PMC2728643

[29] Stalberg K Haglund B Stromberg B Kieler H. Prenatal exposure to medicines and the risk of childhood brain tumor. Cancer Epidemiol. (2010) 34:400–4. 10.1016/j.canep.2010.04.018 20510665

[30] Greenop KR Miller M de Klerk NH Scott RJ Attia J Ashton LJ . Maternal dietary intake of folate and vitamins B6 and B12 during pregnancy and risk of childhood brain tumors. Nutr Cancer. (2014) 66:800–9. 10.1080/01635581.2014.916326 24897174

[31] Bhatti P Doody DR Mckean-Cowdin R Mueller BA. Neonatal vitamin D and childhood brain tumor risk. Int J Cancer. (2015) 136:2481–5. 10.1002/ijc.29291 25348494 PMC4355103

[32] Greenop KR Miller M Bailey HD de Klerk NH Attia J Kellie SJ . Childhood folate, B6, B12, and food group intake and the risk of childhood brain tumors: Results from an Australian case-control study. Cancer Causes Control. (2015) 26:871–9. 10.1007/s10552-015-0562-z 25791129

[33] Zigmont V Garrett A Peng J Seweryn M Rempala GA Harris R . Association between prediagnostic serum 25-Hydroxyvitamin d concentration and glioma. Nutr Cancer. (2015) 67:1120–30. 10.1080/01635581.2015.1073757 26317248 PMC4827350

[34] Huang J Weinstein SJ Kitahara CM Karoly ED Sampson JN Albanes D . prospective study of serum metabolites and glioma risk. Oncotarget. (2017) 8:70366–77. 10.18632/oncotarget.19705 29050286 PMC5642561

[35] Heydari M Shayanfar M Sharifi G Saneei P Sadeghi O Esmaillzadeh A. The association between dietary total antioxidant capacity and glioma in adults. Nutr Cancer. (2021) 73:1947–56. 10.1080/01635581.2020.1817954 32912000

[36] Yue Y Creed JH Cote DJ Stampfer MJ Wang M Midttun O . Pre-diagnostic circulating concentrations of fat-soluble vitamins and risk of glioma in three cohort studies. Sci Rep. (2021) 11:9318. 10.1038/s41598-021-88485-0 33927267 PMC8084971

[37] Metodiewa D Koska C. Reactive oxygen species and reactive nitrogen species: Relevance to cyto(neuro)toxic events and neurologic disorders. Overv Neurotox Res. (2000) 1:197–233. 10.1007/BF03033290 12835102

[38] Hung CF Lu KH. Vitamin C inhibited DNA adduct formation and arylamine N-acetyltransferase activity and gene expression in rat glial tumor cells. Neurochem Res. (2001) 26:1107–12. 10.1023/a:1012314705007 11700952

[39] Hervouet E Staehlin O Pouliquen D Debien E Cartron PF Menanteau J . Antioxidants delay clinical signs and systemic effects of ENU induced brain tumors in rats. Nutr Cancer. (2013) 65:686–94. 10.1080/01635581.2013.789541 23859036

[40] Yiang GT Chen TY Chen C Hung YT Hsueh KC Wu TK . Antioxidant vitamins promote anticancer effects on low-concentration methotrexate-treated glioblastoma cells via enhancing the caspase-3 death pathway. Food Sci Nutr. (2021) 9:3308–16. 10.1002/fsn3.2298 34136195 PMC8194871

[41] DeLorenze GN McCoy L Tsai AL Quesenberry CJ Rice T. Il'Yasova D . Daily intake of antioxidants in relation to survival among adult patients diagnosed with malignant glioma. Bmc Cancer. (2010) 10:215. 10.1186/1471-2407-10-215 20482871 PMC2880992

[42] Ueno M Inano H Onoda M Murase H Ikota N Kagiya TV . Modification of mortality and tumorigenesis by tocopherol-mono-glucoside (TMG) administered after X irradiation in mice and rats. Radiat Res. (2009) 172:519–24. 10.1667/RR1695.1 19772473

[43] Liu RZ Li S Garcia E Glubrecht DD Poon HY Easaw JC . Association between cytoplasmic CRABP2, altered retinoic acid signaling, and poor prognosis in glioblastoma. Glia. (2016) 64:963–76. 10.1002/glia.22976 26893190 PMC5595534

[44] Aggarwal S Subberwal M Kumar S Sharma M. Brain tumor and role of beta-carotene, a-tocopherol, superoxide dismutase and glutathione peroxidase. J Cancer Res Ther. (2006) 2:24–7. 10.4103/0973-1482.19771 17998669

[45] Wang CJ Lin JK. Inhibitory effects of carotenoids and retinoids on the in vitro growth of rat C-6 glioma cells. Proc Natl Sci Counc Repub China B. (1989) 13:176–83. 2480612

[46] Terry MB Howe G Pogoda JM Zhang FF Ahlbom A Choi W . An international case-control study of adult diet and brain tumor risk: a histology-specific analysis by food group. Ann Epidemiol. (2009) 19:161–71. 10.1016/j.annepidem.2008.12.010 19216998 PMC3832293

[47] Sirachainan N Wongruangsri S Kajanachumpol S Pakakasama S Visudtibhan A Nuchprayoon I . Folate pathway genetic polymorphisms and susceptibility of central nervous system tumors in Thai children. Cancer Detect Prev. (2008) 32:72–8. 10.1016/j.cdp.2008.02.004 18406541

[48] Liu H Sun Q Zhang M Zhang Z Fan X Yuan H . Differential expression of folate receptor 1 in medulloblastoma and the correlation with clinicopathological characters and target therapeutic potential. Oncotarget. (2017) 8:23048–60. 10.18632/oncotarget.15480 28416738 PMC5410284

[49] Guo J Schlich M Cryan JF O'Driscoll CM. Targeted drug delivery via folate receptors for the treatment of brain cancer: Can the promise deliver? J Pharm Sci. (2017) 106:3413–20. 10.1016/j.xphs.2017.08.009 28842300

[50] Hervouet E Debien E Campion L Charbord J Menanteau J Vallette FM . Folate supplementation limits the aggressiveness of glioma via the remethylation of DNA repeats element and genes governing apoptosis and proliferation. Clin Cancer Res. (2009) 15:3519–29. 10.1158/1078-0432.CCR-08-2062 19451595

[51] Okada M Suzuki S Togashi K Sugai A Yamamoto M Kitanaka C. Targeting folate metabolism is selectively cytotoxic to glioma stem cells and effectively cooperates with differentiation therapy to eliminate Tumor-Initiating cells in glioma xenografts. Int J Mol Sci. (2021) 22:1633. 10.3390/ijms222111633 34769063 PMC8583947

[52] Lo CS Kiang KM Leung GK. Anti-tumor effects of vitamin D in glioblastoma: Mechanism and therapeutic implications. Lab Invest. (2022) 102:118–25. 10.1038/s41374-021-00673-8 34504307

[53] Baudet C Perret E Delpech B Kaghad M Brachet P Wion D . Differentially expressed genes in C69 glioma cells during vitamin D-induced cell death program. Cell Death Differ. (1998) 5:116–25. 10.1038/sj.cdd.4400327 10200452

[54] Said AK Essien EE Abbas M Yu X Xie W Sun J . Association between dietary nitrate, nitrite intake, and site-specific cancer risk: a systematic review and meta-analysis. Nutrients. (2022) 14:666. 10.3390/nu14030666 35277025 PMC8838348

[55] Essien EE Said AK Cote A Mohamed KS Baig M Habib M . Drinking-water nitrate and cancer risk: a systematic review and meta-analysis. Arch Environ Occup Health. (2022) 77:51–67. 10.1080/19338244.2020.1842313 33138742

