# Supplementary material for: Association between vitamins and risk of brain tumors: A systematic review and dose-response meta-analysis of observational studies
Source: Front Nutr. 2022 Jul 29;9:935706. doi: 10.3389/fnut.2022.935706 (PMC9372437; doi:10.3389/fnut.2022.935706)
Supplement: Supplementary file 1 [file Data_Sheet_1.docx]

Supplementary Material


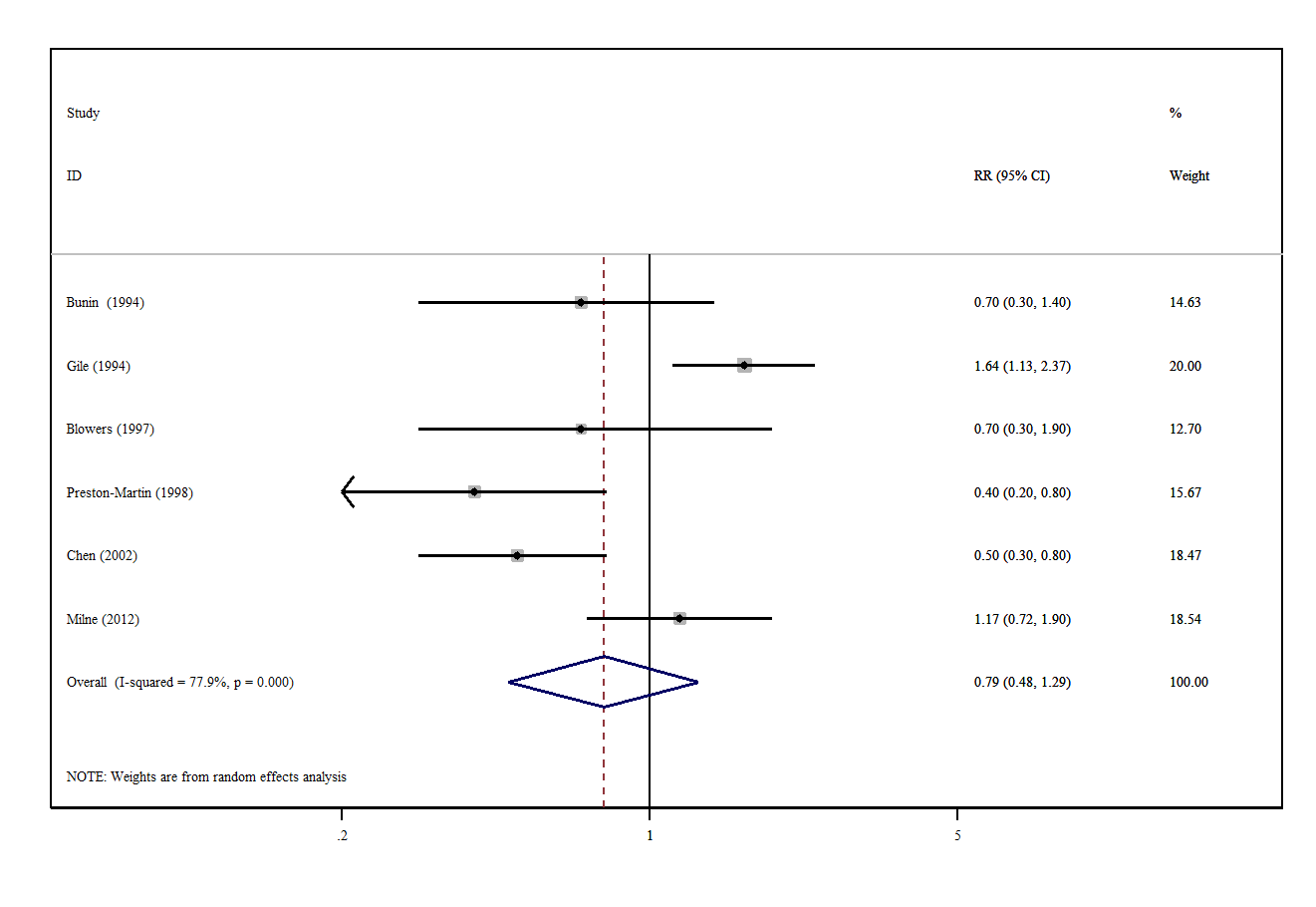


**Supplementary Figure 1** **|** A forest plot showing risk estimates of the association between vitamin A intake and brain tumors.


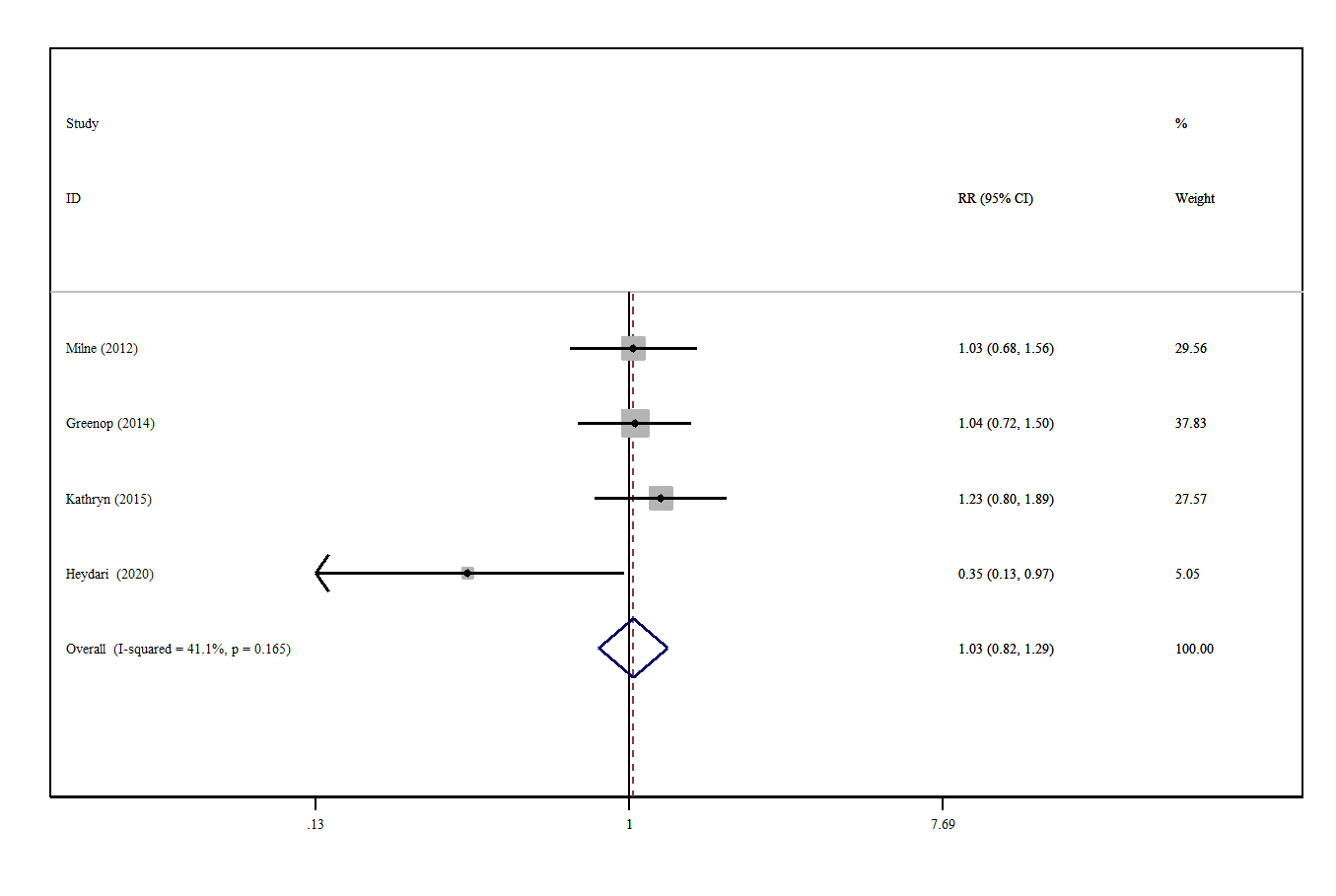


**Supplementary Figure 2 |** A forest plot showing risk estimates of the association between vitamin B intake and brain tumors.


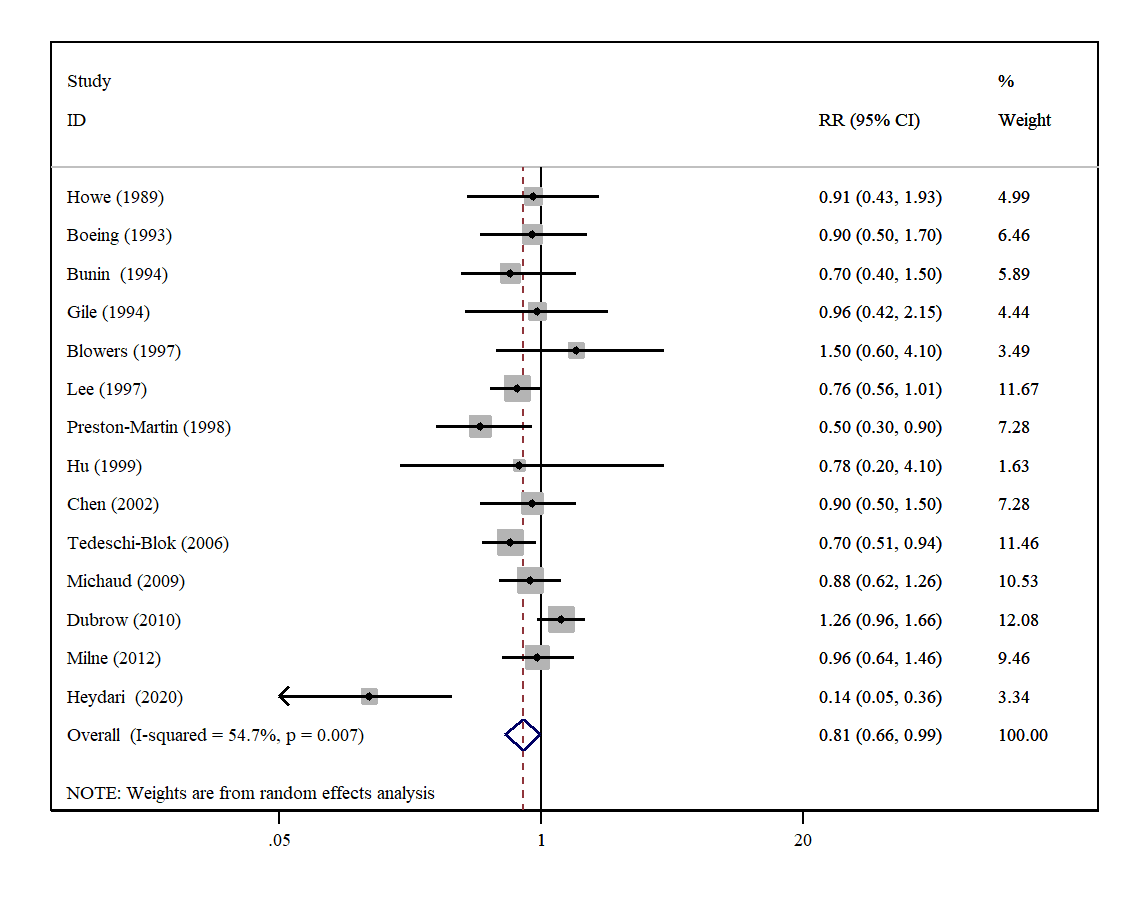


**Supplementary Figure 3** **|** A forest plot showing risk estimates of the association between vitamin C intake and brain tumors.


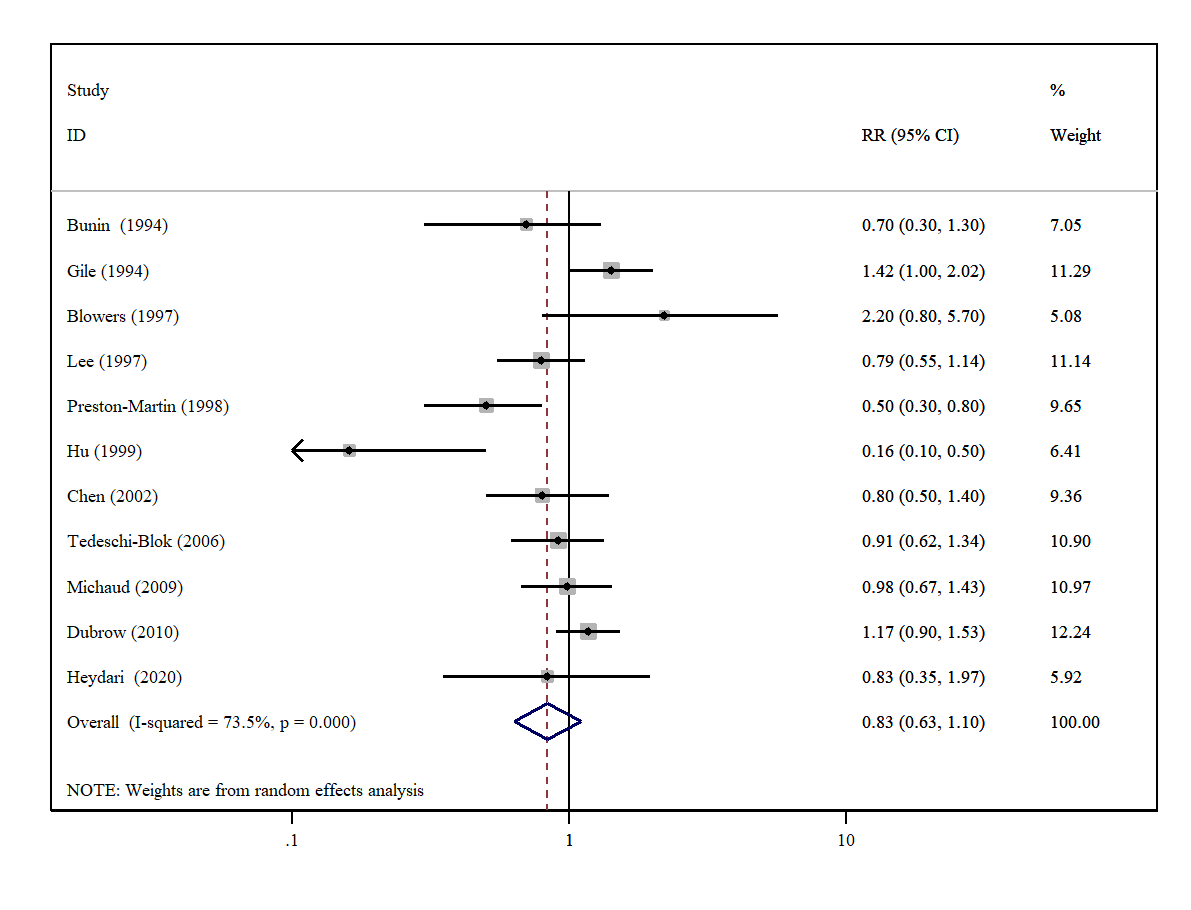


**Supplementary Figure 4 |** A forest plot showing risk estimates of the association between vitamin E intake and brain tumors.


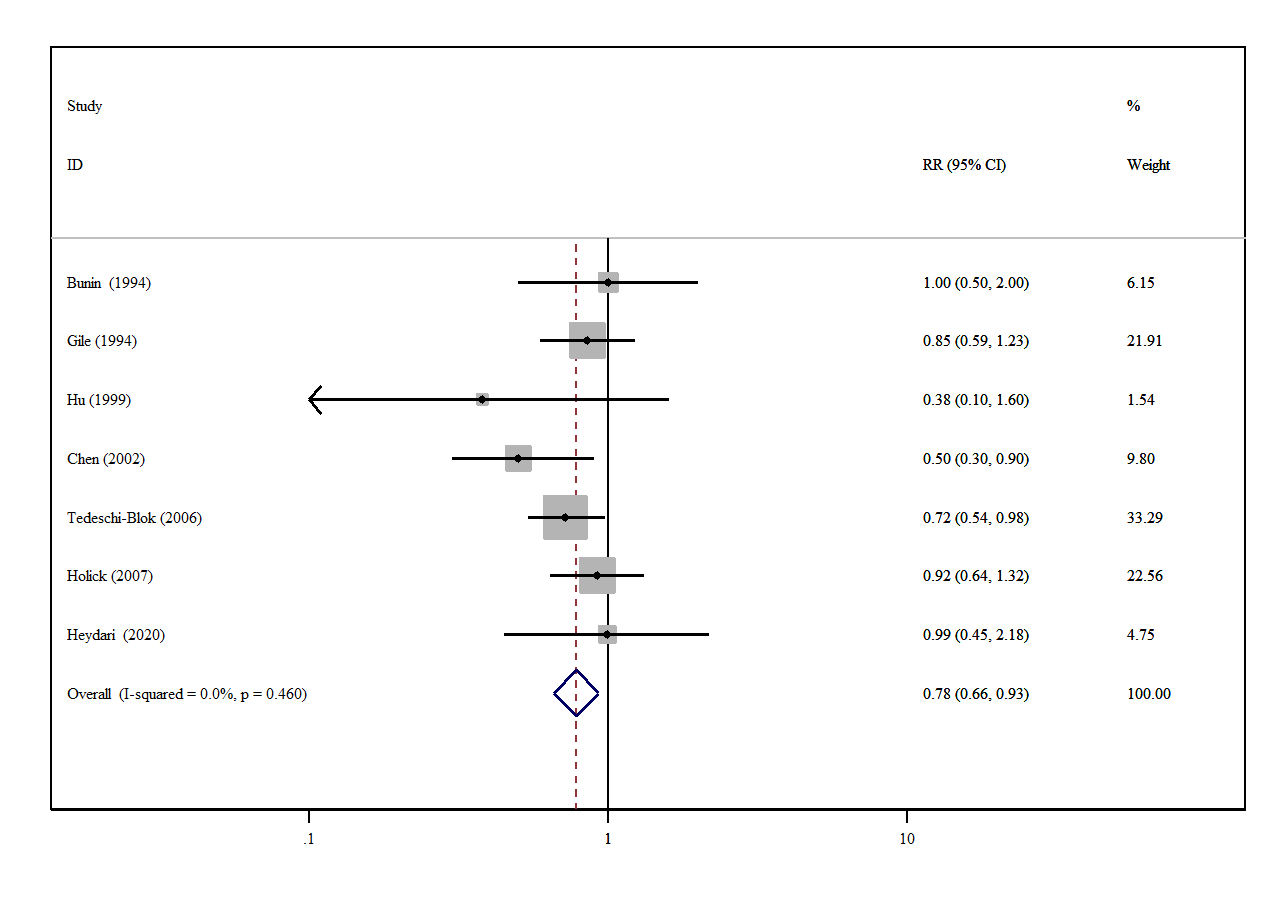


**Supplementary Figure 5 |** A forest plot showing risk estimates of the association between β-carotene intake and brain tumors.


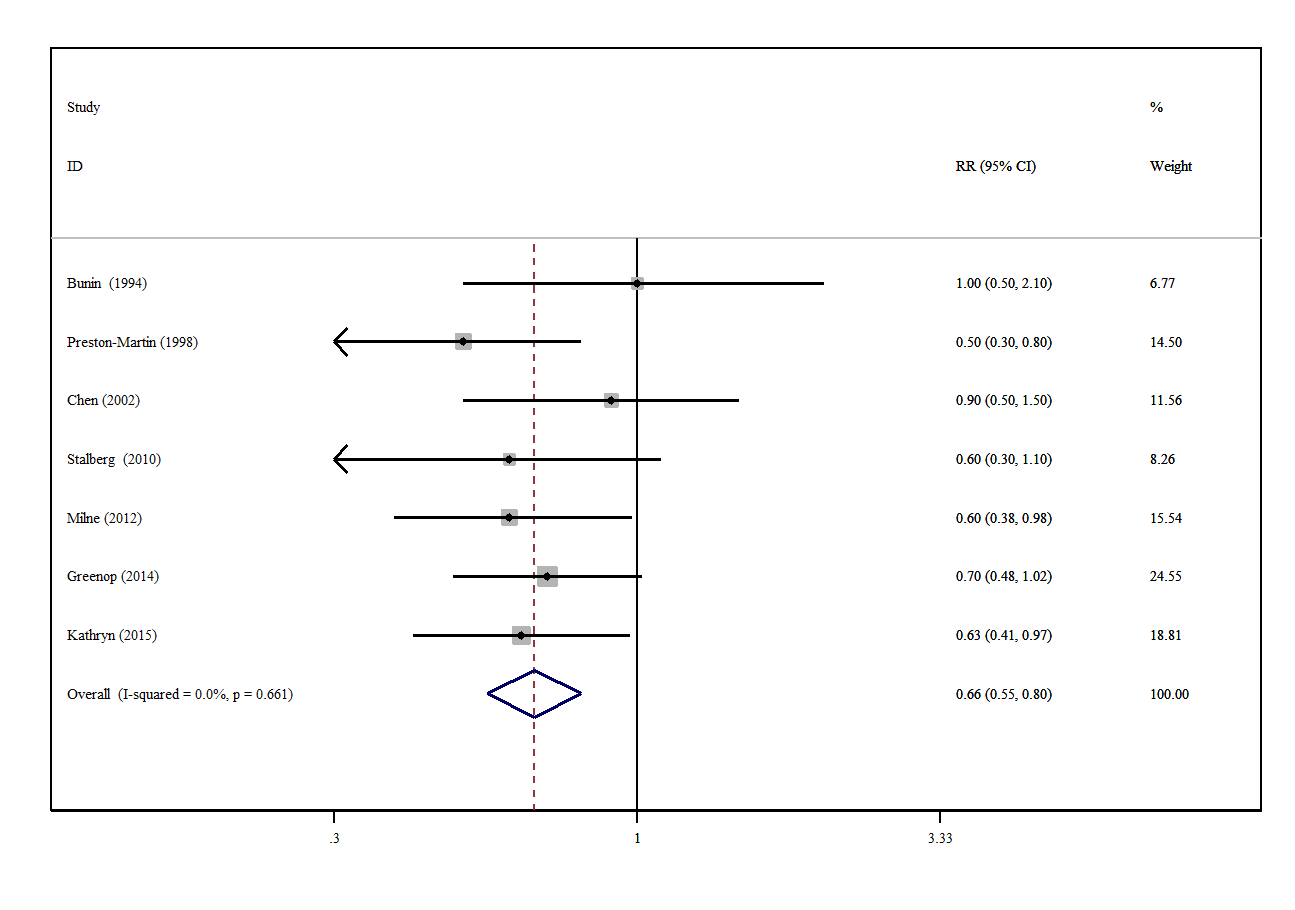


**Supplementary Figure 6 |** A forest plot showing risk estimates of the association between folate intake and brain tumors.


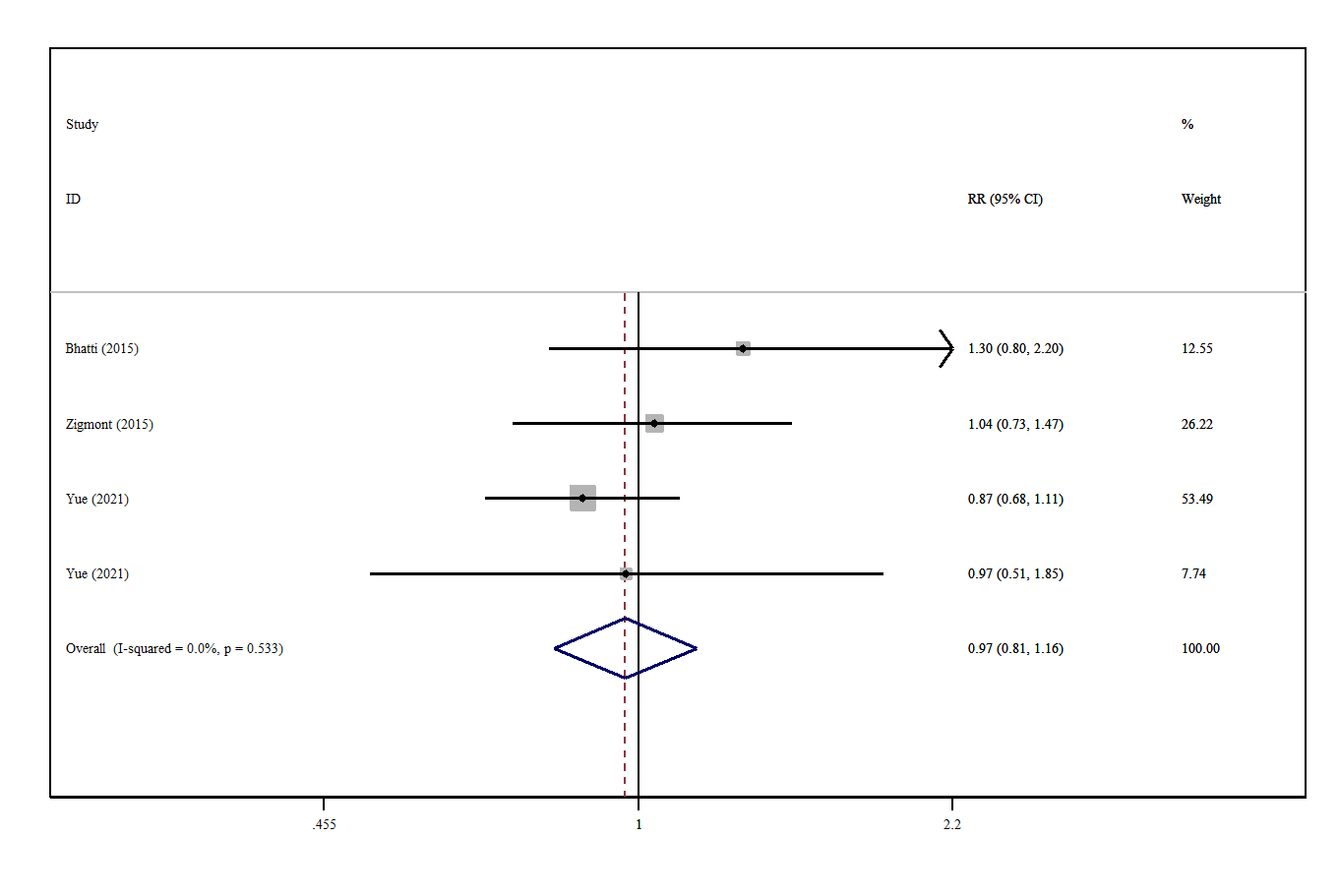


**Supplementary Figure 7 |** A forest plot showing risk estimates of the association between serum 25-hydroxyvitamin D concentration and brain tumors.


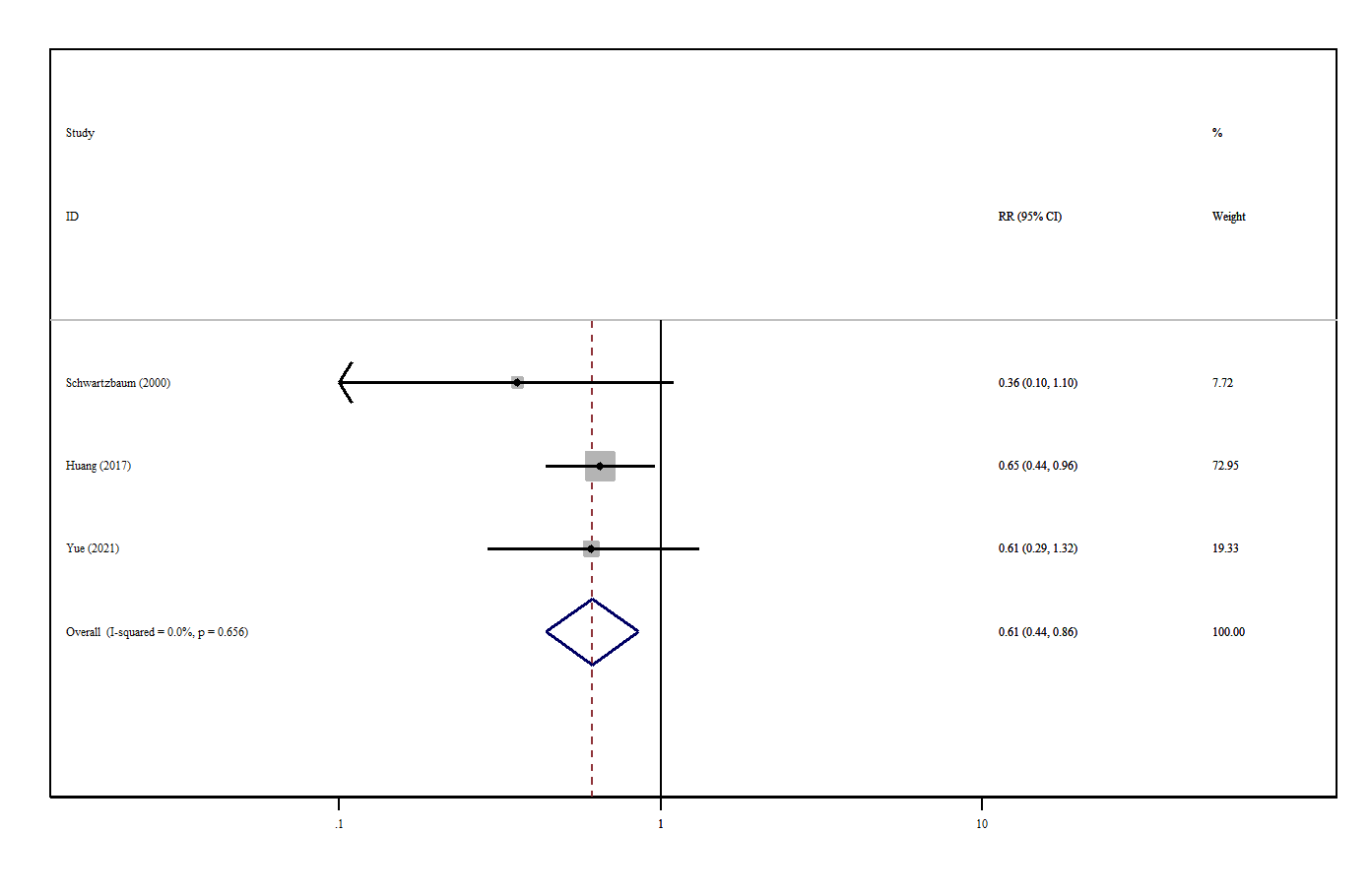


**Supplementary Figure 8 |** A forest plot showing risk estimates of the association between serum α-tocopherol concentration and brain tumors.
